# Descriptive Analysis of Physical Activity Initiatives for Health Promotion in Saudi Arabia

**DOI:** 10.3389/fpubh.2018.00329

**Published:** 2018-11-14

**Authors:** Hazzaa M. Al-Hazzaa, Mezna A. AlMarzooqi

**Affiliations:** ^1^Lifestyle and Health Research, Health Science Research Center, Princess Nourah Bint Abdulrahman University, Riyadh, Saudi Arabia; ^2^Department of Community Health Sciences, College of Applied Medical Sciences, King Saud University, Riyadh, Saudi Arabia

**Keywords:** physical activity, physical inactivity, physical activity initiatives, physical activity promotion, sedentary lifestyle, Saudi Arabia

## Abstract

**Background:** Although the benefits of physical activity are well acknowledged, a high percentage of Saudi population, especially females, remain essentially physically inactive. Getting inactive people to start participating in physical activity and to keep exercising remains a great challenge. Physical activity initiatives in the country have not been previously documented. Therefore, the aim of this article was to provide a narrative review of the physical activity initiatives and discusses influencing factors.

**Methods:** Publically-available physical activity initiatives conducted before June 2018 were searched through the web or they were obtained straight from the organization themselves. The search focus was on any initiative aimed to promote physical activity and mass sports participation and encourage people to adopt active living habit.

**Results:** Numerous initiatives aimed at promoting physical activity existed in Saudi Arabia. However, a common attribute of these initiatives is that they were fragmented, short term attempts, and lacked a coordinating body. The majority of the physical activity initiatives also lacked objective evaluations of their outcomes. It was clear that more physical activity opportunity must be provided for Saudi girls, women, and elderly. There is a need for establishing a national policy encouraging active living and discouraging sedentary lifestyle with contributions from all involved parties.

**Conclusions:** Based on the available evidences, more intensified efforts toward promoting physical activity and reducing sedentary behaviors among Saudi population are needed in order to reduce the risks of NCD's.

## Background

Physical activity (PA) is a health behavior that is well recognized for its role in promoting health and preventing disease (1–3). On the other hand, physical inactivity and sedentary lifestyle are associated with many undesirable health outcomes, like weight gain, obesity, reduced cardiorespiratory, and musculoskeletal function, less favorable metabolic health, insulin resistance and type 2 diabetes mellitus and decreased cognitive function (1, 4, 5). It is well recognized that physical inactivity is a significant contributor to non-communicable diseases in high income countries, and is gradually becoming so in those countries with low and middle income (1, 6). Indeed, physical inactivity alone is reported to be responsible for 9% of global premature mortality, or more than 5.3 million deaths annually (1). In addition to morbidity and premature mortality, physical inactivity appears to be responsible for a considerable economic burden worldwide, amounting to US$ 53.8 billion in 2013 (7). This has lead World Health Organization (WHO), in its recent report, to target a 15% relative reduction in the global prevalence of physical inactivity in adults and in adolescents by the year 2030 (3).

The Kingdom of Saudi Arabia has witness in recent decades an enormous economic development accompanied by modernization, lifestyle transformation, rapid demographic changes, and extensive urbanization. This has led to major negative changes in the people's lifestyle behaviors, with increased prevalence of physical inactivity and sedentary behaviors among Saudi society (8–10). Such negative lifestyle behaviors contributed greatly to a rise in lifestyle-related NCD's in the country including obesity, diabetes mellitus, coronary artery diseases, and hypertension (11–13). However, despite large and strong body of evidence supporting the role of PA in preventing chronic non-communicable diseases (NCDs), a high percentage of Saudi population remain essentially physically inactive (14). Among female population, in particular, recent researches showed that nearly 78% of Saudi adult women (10) and 78.1% of Saudi adolescent females were inactive (9). Consequently, physical inactivity in Saudi Arabia represents a public health burden. In fact, population attributable fraction (PAF) for all-cause mortality associated with physical inactivity in Saudi Arabia reached 18.4% and can be greatly eliminated if all inactive people in the country engage in the recommended PA (1). Such PAF appears much higher than the median for Eastern Mediterranean Region (12.5%) or the median for all WHO regions (9.4%) (1).

In Saudi Arabia, it was estimated that the direct and indirect cost of physical inactivity in 2013 to be 1.71% of the total health-care cost in the country (7). In order to promote active living, reduce physical inactivity and reduce the health cost incurred by inactivity in the country, concerted efforts need to be stepped up and likely barriers to PA must be determined and eliminated. According to World Health Organization report, a number of barriers related to social, cultural and built-environment are believed to be attributed to global inactivity epidemic. Such factors include fear of violence and crime, high density traffic, low air quality and lack of appropriate exercise places, sports facility, sidewalks, and parks (15). Additional factors that are believed to be negatively associated with adult physical activity include advancing age, low income, lack of time, low motivation, and poor health (16). Family income may also influence physical inactivity, as those with lower income appear less likely to meet PA guidelines when compared to higher income people (17).

A recent WHO report showed that policies to address insufficient PA are operational in only half of WHO member countries and that around quarter of adults aged 18 and older were not engaging in health-enhancing PA, with more inactive women than men (3, 15). Indeed, WHO revealed that the most health challenges facing Saudi Arabia are increases in non-communicable diseases (NCDs) and associated modifiable risk factors, including physical inactivity, unhealthy dietary habits, and tobacco smoking (18). The recent Saudi Arabia Ministry of Health Initiatives (19) and Saudi Vision 2030 (20) acknowledged these challenges and have stressed the importance of healthy lifestyle in improving the health and prosperity of all segments of Saudi population. A common strategy that can be pursued in this regard is to address chronic disease prevention through active living initiatives intend to promote PA and reduce sedentary lifestyle.

Although the benefits of PA are well acknowledged, getting inactive people to start participating in PA and to keep exercising remains a great challenge. Therefore, Saudis need to create ways to make PA opportunities more available in schools, workplaces, and within the community settings. However, PA initiatives for promoting active living and healthy lifestyles in the country have not been previously documented. It is anticipated that this review will be a base document contributing to the future implementations of the country health plans and strategy aiming on combating physical inactivity and encouraging active lifestyle among Saudi population. Therefore, the aim of this article was to provide a narrative review of the PA initiatives, which have been conducted in the last two decades, and intended to promote active living among Saudi population. It was also the intent of this paper to discuss factors that influence the sustainability of such initiatives.

## Materials and Methods

Since there is a lack of published literature regarding PA promotional initiatives in the country, it was not possible to conduct a systematic literature review on this topic. Therefore, less formal search was conducted, using publically-available PA initiatives organized between January 2000 and June 2018 that were searched through the web or such information was directly obtained from the organizations themselves. The search keys that were used included the following topics: physical activity initiatives and Saudi Arabia; physical activity promotion and Saudi Arabia; sport participation initiatives and Saudi Arabia; movement initiatives and Saudi Arabia; physical activity strategy and Saudi Arabia; and promoting active living and Saudi Arabia. The inclusion criteria for this narrative review included any nationally- or provincially-based initiatives that was sponsored by government, non-profit or private entities with the main emphasis on PA or active living promotion through education, increasing knowledge/awareness about PA benefits or attempting to reverse the sedentary behaviors of the Saudi people through physical activity initiatives. The target audience could have been the general public or specific groups (e.g., schools' students, teachers, patients, healthcare providers or women). Such initiatives may include promoting mass participation in PA and sports, encouraging healthy lifestyle through active living and environmentally-based initiatives intended to improve healthy living or reduce risks of chronic diseases through engaging in PA. We have also categorized such initiatives into seven sectors, including health, education, sport, urban design, environment, transport, and tourism.

## Results

The search yielded 10 major initiatives for the promotion of PA that have been conducted in the country (Table 1). These initiatives were implemented by many national, and provincial institutions and organizations that are involved in exercise and PA promotion. Such initiatives covered wide range of sectors, including health, education, sports, urban design, environment, transportation, and tourism. The institutions responsible for the promotion of PA include mainly Ministry of Health (MOH), Ministry of Education (MOE), Saudi Sports Authorities, Ministry of Municipality affairs, and Ministry of Planning. Other organizations may also be involved by different means include sports sciences departments in the universities, sports medicine association, sports for all federation, Saudi heart association, Saudi obesity association, Saudi nutrition society, and Saudi physical therapy association. At the sports and competitive levels, involved organizations include Saudi Arabian National Olympic Committee (SAOC), sports federations, sports clubs, and athletic/sports departments at colleges and universities around the countries. In addition, numerous events and initiatives are carried out throughout the country by various organizations and institutions during World Health Day and Physical Activity Day, which are mainly intended for increasing awareness about health benefits of PA and healthy lifestyle.

**Table 1:** Saudi Arabian major health and physical activity initiatives by sectors.

**Physical activity initiatives**	**Sector**						
	**Health**	**Education**	**Sport**	**Urban design**	**Environment**	**Transport**	**Tourism**
1. The Healthy City Initiative	✓			✓	✓		
2. Ministry of Health initiatives	✓	✓					
3. Ministry of Education Initiatives	✓	✓					
4. The Municipalities' Initiatives				✓	✓	✓	✓
5. The high Commission for the Development of Riyadh			✓	✓	✓		
6. The Saudi Arabian Olympic Committee and Sports Federations	✓		✓				
7. The Al-Haraka Baraka PA promotional Initiative	✓	✓					
8. Strategy to Combat Obesity and Promote PA in the Arab Countries	✓	✓					
9. Saudi Arabia is Walking Initiative	✓						
10. The National Transformation Program in Vision 2030	✓	✓	✓		✓	✓	

The following sections have more detailed description of major health and physical activity initiatives along with the organizations that are responsible for conducting such initiatives.

## The Healthy City Initiative

The Healthy City Initiative was first presented by the World Health Organization, Eastern Mediterranean Region (21). It was introduced in Saudi Arabia in 1999. The program was initially implemented in one city but has since been expanded to cover 25 cities. It supports a range of activities, including health promotion, environmental health issues, safe and friendly streets and communities for walking as well as skills development for women and youth. The main challenges for the healthy city program in Saudi Arabia were rapid urbanization and population growth in most of cities, due to migration to the city from rural areas, a high number of road traffic injuries and less favorable air quality.

## Ministry of Health Initiatives

The Ministry of Health (MOH), has assumed a leading leadership role in recent years in providing initiatives and programs for health-enhancing PA around the country. The MOH has introduced many initiatives and programs that are intended for educating and training primary health care professionals about PA and exercise prescription for patients. Moreover, as part of the National Strategy on Diet and Physical Activity, the MOH has held for the last seven years numerous lectures, workshops, and training courses in many major cities of the country on the subject of balanced diet and PA. These activities served in educating and training health care professionals with knowledge and skills that are necessary to prescribe healthy diet and PA for patients. They have also produced training manuals on balanced diet and PA, brochures and posters, created an electronic educational site and conducted media awareness campaigns. The MOH is also working on national strategy for PA. Additionally, among the MOH projects, was the execution of the PA surveys during the year 2005, and again in 2013 and in 2017 as part of national health survey. Further, *Sahatik Beddonia*, which means “Your Health should worth everything to you,” is another promotional program that was implemented by the Non-communicable Disease branch of the MOH, targeting weight loss through proper diet and PA. The programs are also available at many social media links (22, 23).

## Ministry of Education Initiatives

For a long time, the Ministry of Education (MOE), through the school health program, has established a program called health-promoting schools, which is widely implemented in many schools around the country, especially in recent years. Also, among the initiatives of the MOE is the health-enhancing PA program at public schools. Recently, King Abdullah program for Education Development (*Tatweer*) has initiated the School Sports Strategy (24), which among other things, aims at providing a platform for increased PA participation in schools. Indeed, a very recent and important initiatives by the MOE has been the officially introduction of the physical education program for the first time for girls' schools. Schools are now able to hold PA classes inside schools beginning in the school year 2017/2018. Such important development, which is intended to enhance good health, physical fitness and total well-being among the girls, may well increase physical activity participation among girls and women in the country.

## The Municipalities' Initiatives

The Municipalities around the country have also had many programs and activities aimed at improving the quality of life of the Saudi people, by creating more parks, walking trails and community recreational spaces. In Riyadh city for example, there are mega parks with provisions for walking tracks. In many cities around the country, the municipalities have also initiated what is called “popular playgrounds” within the communities, which have provision for free space with some sports and recreational facilities for people of all ages. Walking trails are increasingly being built by the municipalities all over the cities in Saudi Arabia. However, in many parts of Saudi Arabia, outdoor sports participation especially in the summer months (June to August) is somewhat restricted because of the extreme hot climate, which can reach well above 45°C.

## The high Commission for the Development of Riyadh

The high Commission for the Development of Riyadh city has particularly done a tremendous work in relations to recreational facilities around Riyadh. The Commission has inaugurated several projects in the last 15 years, which includes mega scale parks inside the city and in the surrounding suburbs. These mega scale parks are all equipped with playground for children, walking trials and some with even hiking trials. A mega park project that is worth mentioning is, the *Wadi Hanifah* development and restoration project, which runs over 100 km alongside Riyadh city (25). It was established as an environmental, recreational, and tourism resource, with intentions such as, reinstating its natural beauty, harnessing, and rehabilitating its water and flood performance, thereby, establishing a platform for clean environment, accessibility, public landscape, and cultural resources. This is a good example of a project that is mainly intended for the restoration of the natural environment, because it utilizes a big valley which serves as a recreational area, as well as for the promotion of PA for the whole community. The project even won Aga Khan Award for Architecture. The commission is also responsible for running the beautiful diplomatic quarter west of Riyadh, which has many opportunities for PA including several walking trails and huge aquatic sports facility.

## The Saudi Arabian Olympic Committee and Sports Federations

Over the years, the Saudi Arabian Olympic Committee (26), has been keen on the organization of many sporting events, including those intended for mass participation. Recently, the SAOC has funded and supervised a large project for establishing and implementing a Sports Strategy for Saudi Arabia, including a nationwide survey related to sport and PA participation levels among Saudi people. Moreover, Saudi Federation of Sports Medicine (27), and Saudi Sports for All Federation (28), regularly held numerous lectures, workshops and training courses over the past decades on issues related to PA, fitness and health as well as disseminating many brochures about exercise, PA and health benefits for all ages. In addition, the Leadership Institute within the Saudi Sports Authority constantly administers training courses and workshops on the subjects of PA and fitness targeting coaches and fitness specialists.

## The *Al-Haraka Baraka* physical activity promotional Initiative

In the year 2005/2006, a collaborative effort between a public university (King Saud University), a non-profit organization (Arab Nutrition Center), and private sectors (Mars Middle East Inc.), has led to the initiating of the “*Al-Haraka Baraka*” (AHB) PA program at many schools in the Gulf Cooperation Council (GCC) countries, including Saudi Arabia (29). AHB means “movement is a blessing.” This educational program provides health-enhancing PA lessons and knowledge to school children between the ages of 6 and 12 years. The program provided materials for school's physical educators as well. The AHB program complements and does not compete with the regular school's physical education curriculum. The program has been very popular for several years until it was stopped because of lack of continued funding. It is worth mentioning that the AHB program was subjected to pilot testing and evaluation by the developers. The evaluation was based on students', their family and schools teachers' attitudes, and satisfaction with the program materials and activities.

## Strategy to Combat Obesity and Promote Physical Activity in the Arab Countries

Another important health promotion initiative is the “Strategy to Combat Obesity and Promote Physical Activity in the Arab Countries,” including Saudi Arabia (30). This strategy was approved of by the Arab task Force on Obesity Prevention and PA Promotion meeting, which was held in Bahrain in the year 2010. The strategy was then sent to all Arabs' Health Ministers. It provides useful guidelines for each Arab country to prepare its own strategy and action plan, with an aim to prevent and control obesity, and to promote PA and healthy eating. The strategy focused on expected outcomes, objectives, indicators to measure the objectives and actions needed to implement the strategy in nine target areas. These target areas included child-care centers/preschools, schools, universities, primary health care, secondary care, food companies, food preparation institutes, media, public benefit organizations, and the workplace. Follow-up and future developments of this strategy were also included.

## “Saudi Arabia is walking” Initiative

This is a mass participation campaign, a twitter-based initiative started in 2017 and continued in the year 2018 (31). It originated from a walking group in AlJouf region in northern Saudi Arabia and resonated into other major cities of the country. It aims to increase the number of people males and females who assume regular walking habit for the promotion of health and preventing of chronic disease. In its second year, the initiative get support from the Sports for All Federation. Now, there are more than 50 walking groups in many cities around the country joined this initiative and the number appears growing. In addition, a number of celebrities are also promoting this initiative, which may add momentum to it. Indeed, at some point of time the initiative hashtag reach a trend.

## Saudi Arabia National Transformation Program of the Vision 2030

The Vision 2030 included an ambitious healthy lifestyle promotion programs targeting Saudi population (20). Indeed, level 2.2.1 objectives of the Vision 2030 indicated that “increasing public participation in PA and sports” as an important endeavors to be achieved. In addition, level 2.1.3 objectives explicitly state “strengthen prevention against health threat.” Physical inactivity and other unhealthy lifestyle behaviors are considered major threats to the health of the Saudi population (being major risks for NCD's).

## Discussion

The first formal attempt to examine PA initiatives in the country was conducted in 2013/2014, and the findings were presented in a meeting organized by the WHO Eastern Mediterranean Region in Dubai (32). Based on the present review, it appears that there was not a shortage of PA initiatives, which are intended to promote active living in Saudi Arabia. However, what was clear about these initiatives is that they were somewhat fragmented and appear mostly as short term attempts to promote PA participation. In addition, comprehensive and detailed information about such initiatives were difficult to obtain. Further, there had been little central coordination of these initiatives. Perhaps, there may be a need to establish a coordinating body responsible for the synchronization and harmonization of these initiatives and activities. Moreover, except for a few of these activities, there had been no formal assessment or evaluation carried out for these initiatives and programs.

The MOH, MOE, municipalities, Sports Authority, universities, walking societies, and active community groups are all involved in conducting short terms PA initiatives, including mass participation campaigns. However, MOH PA promotional initiatives seem to be more sustained, systematically organized, diverse and with long-tern goals (e.g., organizing regular training courses for health care providers and promoting health messages/educational materials about PA as well as initiating PA guidelines for the population). It is also clearly evident that most PA initiatives conducted in the country appear to lack inputs and feedbacks from experts in the field of PA promotion, PA epidemiology or behavioral science.

Furthermore, our findings showed that PA initiatives directed toward seniors and challenged people are lacking, especially when knowing that Saudi elderly population is increasing in recent years due to improvement in health care. PA guidelines for adults recommend engaging in at least 150 min of moderate intensity aerobic activity or 75 min of vigorous-intensity PA per week (2). However, recent evidence has challenged this threshold-centered recommendation and suggested that such relationship appears generally curvilinear, meaning that marked health benefits are observed with relatively minor increase in the amount of PA (33). Studies involving older people confirmed that a lower amount of PA can be beneficial and reduces all-cause mortality (33–35).

Moreover, there exist minimal PA initiatives and promotional activity programs that are directed toward workplace in Saudi Arabia. The potential value for the workplace as a useful setting for PA promotion and sedentary behaviors prevention cannot be overstated. Initiatives for promoting and encouraging access to active transportation by means of safe and attractive infrastructures appears lacking. However, mass transportation projects in some major cities of the country are undergoing right now. Additionally, municipalities can go beyond creating walking paths and building parks and paly grounds for local community by implementing low cost sports facilities near residential neighborhoods, such as swimming pools, low cost indoor gym, tennis courts, and mini soccer fields.

The majority of the PA initiatives appeared to be based on the social marketing theory, as mass communication, mass participation, community PA events and sport rallies are clearly dominant in these initiatives. However, there is also a need to focus on the social determinants of health, environment-based interventions as well as embracing a more multi-level approach. Further, it is well recognized that PA programs that focus on behavior change may be of limited effectiveness when structural barriers are not addressed (36). Riyadh declaration on Healthy Lifestyles and Non-communicable Disease in the Arab World and the Middle East called for provisions, when planning new residential developments, to include environments which promote walking or biking, social gathering, and safe space to allow PA for the whole community including women, elderly persons and children (37). Environmental and societal correlates have been identified as important factors to be considered when promoting healthy food habits and regular PA (38). A health friendly environment, however, does not necessarily by itself prevent sedentary lifestyles. Other approaches including the establishing of PA policies, government legislation and action plans, intervention strategies, well designed physical activity programs, national and local campaigns are all need to be explored, with full engagement from relevant societies and official organizations.

Regular PA offers numerous well recognized health benefits leading to enhancement of physical, mental, and social functions at all age levels (1, 3, 39). It is believed that many chronic disease that are today prevalent in Saudi Arabia are linked to a change from traditional to westernized diet and an increase in physical inactivity and sedentary lifestyle that are becoming prevalent in our society (8, 9, 37). Currently, PA in Saudi Arabia appears to be an underserved public health issue (40). Moreover, since physical inactivity has become an epidemic worldwide, numerous global as well as national initiatives have been conducted in schools, families, and communities to encourage the adoption of healthy lifestyle habits (2, 41). Examples of countries who have had successful PA national initiatives included United States (42). Canada (43), Australia (44), New Zealand (45), and Brazil (46). At international level, the WHO Global Action Plan on Non-communicable Disease Prevention and Control aims to reduce physical inactivity by 10% by 2025 and further relative reduction of 5% by the year 2030 (3). However, attaining such a goal requires leadership and contributions from academic and scientific community along with policy makers and practitioners (39).

Schools play an important role in PA promotion for children and adolescents. The proportion of physical education lesson time that students spend in moderate to vigorous physical activity was shown to be less than optimal for health-enhancement; both locally (47) and globally (48). In Saudi Arabia, there has been many PA initiatives at the school's level including the health-enhancing PA program. The promotion of PA in school's setting would certainly have positive effects on students' academic performance and reduces anxiety and stress as well. Indeed, research has shown that students who were physically active achieve better grades and higher scores on standardized tests, in addition to improving cognitive and academic performance as a result of classroom activity breaks (49). This may be partially explained by the increase in blood flow and released hormones as a result of their engagement in PA, hence, improving memory, concentration, and mood as well as reducing anxiety and stress (50).

Based on data from national survey, Saudi women were shown to be much less active than males (10). Among Saudi adolescents, girls appeared consistently less active and have more sedentary behaviors than boys (9). Moreover, it is interesting to note that Saudi adolescents exhibited contrasting results relative to gender and school type. Boys in public schools were more active than in private schools, whereas the opposite was true for girls (9). In addition, exercise timing, PA patterns and the reasons for being active or inactive appeared to be quite different between adolescent boys and girls (51). These differences between males and females at different sittings must be considered when planning PA promotion for the Saudi population. Up to now, there seems to be a gender difference in the availability of opportunities for PA participation. It was also observed that there were fewer initiatives for girls and women compared with men, although this issue has recently been gaining much attention by the government, as Saudi Sports Authority, and in particular the Sports for All Federation is trying hard to fill this gap and provides more opportunities for females to engage in PA and sports. The phenomena of lower PA opportunity for females, however, does not seem peculiar to Saudi Arabia, as the availability of areas for PA was reported to be higher among American men compared to women (52).

In Saudi Arabia, since there is a segregation of males and females in fitness clubs, it is suggested that Sports Authority must opt for more community based fitness centers for girls and women in close proximity to the primary health care centers, in collaboration with the MOH. Community sports clubs may considerably increase leisure-time PA participation, as reported by a recent Australian study (53). Furthermore, previous researches have documented the usefulness of home-and community-based PA and healthy lifestyle promotion for mothers and daughters in both developed (54) and developing countries (55). Likewise, the effectiveness of low intensity, community-based lifestyle intervention for mothers and young children has also been established (56). Needless to say that in order to reach their goals and capacity, PA programs need to be culturally tailored to the expectations of the intended population while at the same time addressing cultural sensitivity and locally specific inactivity determinants. In addition, personal variables, which have significant impacts on lifestyle behaviors, must also be acknowledged.

Health care providers have an important role to play in promoting PA and healthy lifestyle habits among their patients, by providing routine assessment and regular counseling (57). According to a recent WHO Global Action Plan, it is important for the primary and secondary health- and social-care providers to play a guiding and counseling rule for patients of all ages, in order to help them become more active and prevent the increasing burden of NCDs (3). PA can also be used as a means of increasing rates of rehabilitation and recovery (3). Moreover, health care providers can encourage PA for patients and communities by making PA a vital sign and designing innovative active health care environments (58). However, there is a need to provide the primary care providers with the skills on how to counsel their patients about PA, evaluate their current activity level and offer an exercise prescription for them. Exercise is Medicine tools can be adapted and used for such purpose (58, 59). Strategy based on social cognitive theory (60), such as self-efficacy, can also be utilized to increase PA level of the patients.

Healthcare settings in Saudi Arabia can offer a unique opportunity for counseling young and old adults about PA and active living behaviors. However, a review study addressed PA counseling practice among physicians in primary health care centers in Saudi Arabia (with nearly 50 million yearly visits) concluded that there was a limited data available about PA counseling in primary health care providers (61). However, the opportunity and challenges are still there for much needed counseling, as a local study in south western Saudi Arabia revealed that the percentage of individuals attending health promotion clinics who consumed imbalanced diet and were inactive reached more than 90% of the sample (62). In addition, health care providers in Saudi Arabia, need to be a good role model for their patients. However, physical inactivity has been found to be prevalent in health care providers, especially among physician. One study conducted recently in a large medical city in Riyadh to assess healthy lifestyle habits among health care professionals revealed that only 21.1% of the sample were physically active (63). This may present another hindrance to PA promotion, as those inactive healthcare professionals may not prescribe PA for their patients (7).

Health care institutions can also adopt a comprehensive curriculum that potentially closes the gap in medical schools, residency programs, graduate education, and nursing curricula on topics related to PA and exercise prescription and healthy lifestyle habits. Physicians and other health professionals receive very limited formal training in the PA science and clinical applications of exercise, including obesity prevention, nutrition, lifestyle medicine and PA prescription in health and disease. The incorporation of a well-tested PA module into the residency curriculum was shown to be feasible, efficacious, well received, and easily incorporated into the existing curriculum (64). In Saudi Arabia, a recent survey conducted on primary health care centers in the Eastern province of the country, revealed that only one-third of the health care professional believed that they were well prepared to treat obesity and 83% of the respondents had negative attitudes toward the concept of overweight and obesity (65).

The emergence of electronic health communication has the potential to significantly contribute to the promotion of public health. Indeed, social media seem to gain widespread popularity in recent years and can provide an opportunity to serve as communication platforms for healthy lifestyle promotion. However, findings of a survey on the use and preference of the new electronic media communication channels among PA researchers and practitioners revealed that most of the participants in this study preferred non-social media channels to social media platforms (66). The study also found no significant differences between the responses of researchers and the practitioners and that only about 1% of respondents ranked social media sources as their preferred channels for information on PA (66). In Saudi Arabia, a study conducted to identify Twitter influencer profiles for health promotion in Saudi Arabia showed that out of the 99 most influential Twitter accounts in the country only four influencing accounts were focusing on health promotion (67).

It is believed though that mass media-based promotional campaigns were more likely to influence knowledge, relative to behaviors. Rigorous evaluation of such electronic-based campaigns are also needed. A survey conducted on a group of individuals with responsibility for health-promotion programs showed that the majority of health plans had integrated PA messages into routine services for members and sponsored community races, walks, and health fairs, but few rigorous evaluations have been done on these programs (68).

## Recommendations for Promoting PA in Saudi Arabia

Obviously, getting people to change their life style in relation to exercise habits requires a tremendous effort from those involved in governmental policy, community health, school education, municipalities, sports and recreation authorities, and non-governmental organizations (NGO's). However, the following recommendations for promoting PA may be implicated (8, 32, 47).

1. National policies and legislation initiatives are needed that encourage and promote active lifestyle for the whole country with much emphasis on the provision of PA opportunities for all segments of the society. National PA strategy and action plan for implementing, monitoring and evaluating the goals and outcomes of such strategy is also long overdue. All possible stakeholders should be involved in formulating such strategy, including MOH, MOE, Ministry of Municipalities, Ministry of Planning, Ministry of transportation, Ministry of Islamic Endowments and Guidance Affairs, Ministry of Cultures, Ministry Information (including national TV's, Saudi Press Agency and other mass media), the Sports Authority, business and industry, health insurance, Saudi Arabian National Olympic Committee, sports Federations, sports/athletic clubs and fitness centers and major NGO's.
2. Implementation of strong physical education curricula and innovative instructions that emphasize daily physical education lessons and wide-ranging PA opportunities for all students of both sexes with more focus on enjoyable lifelong PA, while at the same time, meeting the needs and interest of all students, including the disabled, the obese, the low fit, and those with chronic health problems. PA opportunities for students beyond physical education, including (but not limited to) active classroom activity breaks, intramural sports, after school activities, and active recess need to be explored. In addition, schools must discourage the use of physical education classes for other subjects (like math or science) at the end of school semester.
3. Provision for more PA/sports facilities and programs, especially for females. Also, school sports facilities should be made available for community after school hours and in the weekend. Investing in low-cost, culturally appropriate, and weather-controlled community sports/recreation facilities (exercise and sports halls) is also needed.
4. Health care providers and medical societies must assume a leading role in educating the public about the benefits of PA as well as encouraging and advocating active lifestyle and healthy eating habits among the Saudi people. They also have an important role to play in physical activity promotion, by providing routine assessment and counseling on PA and lifestyle issues for their patients.
5. Active approaches requiring individual's initiative (such as enrolling in exercise program or increasing peoples' awareness on the importance of PA), though partially successful in promoting PA, these are not enough. Passive tactics should be incorporated, too. Such strategies include provisions on walking trails, time allowance for exercise at work and having school facilities opened for community use after school hours.
6. Opportunities for PA should be made available for a wide range of people including the elderly, children, and women. For municipalities, this means establishing additional safe and convenient walking, jogging, and bicycling paths, and playgrounds and fitness areas for children and adults. For schools, this may include enabling the community to use facilities on weekend. Schools could also hold health, fitness and PA fair, and invite parents as well. Summer camps for obese or disabled children and adolescents should be fully considered, where they can learn about adapted PA, fitness, and healthy eating.
7. Business establishments can support and promote healthy lifestyle through funding PA initiatives, community fitness programs and mass participation in sports as part of their social responsibility.
8. Innovative ideas and approaches for planning fitness facilities and exercise spaces should be fully explored. This may include creating more shaded pedestrian zones, encouraging walkability in shopping malls and establishing fitness centers in workplaces and community settings. Indeed, having designated walking tracks in major shopping malls may encourage people to engage in regular PA.
9. Colleges and universities should consider establishing innovative programs in PA science, fitness, wellness and health promotion, which can provide trained graduates in such areas as fitness, health and wellness, healthy lifestyle and PA promotion. Training females' specialist in physical activity and health and fitness is also a must.
10. There is a need to develop and promote urban and rural planning regulations that promote and encourage PA and active commuting.
11. The social media and new apps and technology tools can be fully utilized in order to encourage PA participation, enable exercise monitoring and facilitate PA advocacy.
12. There may be a need to encourage and possibly subsidize the establishment of work-place fitness centers, PA and exercise initiatives, health and wellness and active lifestyle programs, especially for inactive women.

## Limitations

The present review has several limitations. First, it may not be comprehensive and inclusive enough to include all those local or small initiatives that spread all over the country beyond the authors' knowledge. Second, our search strategy was limited to initiatives that focused explicitly on PA and active living promotion. Other initiatives that directed toward health promotion in general were not considered. Finally, our search analysis did not attempt to document whether desired outcomes related to the initiatives were achieved, as the majority of these initiatives were not really properly evaluated.

## Summary and Conclusion

The high prevalence of inactivity in Saudi Arabia, particularly among females, represents a major public health burden and a precursor for increased prevalence of numerous chronic diseases. Moreover, there remains a need to further understanding of the personal, social and environmental barriers to PA in Saudi population, particularly in relation to different domain of PA (leisure time, occupational, transports, and households PA). The current review showed that there have been numerous initiatives aimed at promoting PA in Saudi Arabia. However, a common attribute of these initiatives is that they are fragmented, short term attempts, and lacked a central coordination. Further, the majority of the physical activity initiatives without objective evaluations of their outcomes. It was clear that more physical activity opportunity must be provided for Saudi girls, women, and elderly. In addition, it is recommended that a national policy encouraging active living and discouraging sedentary lifestyle be established with participation from all involved parties. It is clear that if PA promotion to be successful and sustained as public health initiatives and as part of the country's Vision 2030, a multidisciplinary approach is needed. For the genuine promotion of PA in the country, partnerships with private and industrial communities are also essential. Finally, health care providers have an important role in promoting PA and encouraging the adoption of healthy lifestyle habits among all Saudi people.

## Author Contributions

HA-H: study concept; HA-H and MA: data collection; HA-H: drafting the paper; MA: revising it critically for intellectual content. Both authors critically read and approved the final version of the manuscript.

### Conflict of Interest Statement

The authors declare that the research was conducted in the absence of any commercial or financial relationships that could be construed as a potential conflict of interest.
